# Where the Aryl Hydrocarbon Receptor Meets the microRNAs: Literature Review of the Last 10 Years

**DOI:** 10.3389/fmolb.2021.725044

**Published:** 2021-10-21

**Authors:** Geonildo Rodrigo Disner, Monica Lopes-Ferreira, Carla Lima

**Affiliations:** Immunoregulation Unit of the Laboratory of Applied Toxinology (CeTICS/FAPESP), Butantan Institute, São Paulo, Brazil

**Keywords:** AhR, miRNAs, immunity, inflammation, toxicology, epigenetics

## Abstract

The aryl hydrocarbon receptor (AhR) is an environmentally responsive ligand-activated transcription factor, identified in the ‘70s for its toxic responses to halogenated polycyclic aromatic hydrocarbons, such as dioxin. Recently, AhR has been recognized as engaged in multiple physiological processes in health and diseases, particularly in the immune system, inflammatory response, tumorigenesis, and cellular differentiation by epigenetic mechanisms involving miRNAs. However, there is still scarce information about AhR-dependent miRNA regulation and miRNA-mediated epigenetic control in pathologies and therapies. In this review, we explore the mutual regulation of AhR and miRNA over the last decade of studies since many miRNAs have dioxin response elements (DRE) in their 3’ UTR, as well as AhR might contain binding sites of miRNAs. TCDD is the most used ligand to investigate the impact of AhR activation, and the immune system is one of the most sensitive of its targets. An association between TCDD-activated AhR and epigenetic mechanisms like post-transcriptional regulation by miRNAs, DNA methylation, or histone modification has already been confirmed. Besides, several studies have shown that AhR-induced miR-212/132 cluster suppresses cancers, attenuates autoimmune diseases, and has an anti-inflammatory role in different immune responses by regulating cytokine levels and immune cells. Together the ever-expanding new AhR roles and the miRNA therapeutics are a prominent segment among biopharmaceuticals. Additionally, AhR-activated miRNAs can serve as valuable biomarkers of diseases, notably cancer progression or suppression and chemical exposure. Once AhR-dependent gene expression may hinge on the ligand, cell type, and context singularity, the reviewed outcomes might help contextualize state of the art and support new trends and emerging opportunities in the field.

## Highlights

• AhR research has shifted to new AhR functions beyond toxicology.
• AhR binds to many exogenous ligands such as xenobiotics or endogenous ligands, or even produced by the body or microbiota.
• AhR signals through epigenetic mechanisms control development, apoptosis, immune response, and oncogenesis.
• AhR-mediated miRNA expression depends on the ligand, cell, species, and context peculiarity.
• Most of the AhR-related miRNAs are dependent on AhR activation.
• miRNAs also control the expression of AhR, ARNT, and AhRR.

## Introduction

The aryl hydrocarbon receptor (AhR) is a cytosolic ligand-dependent transcription factor and environmental sensor which belongs to the basic helix-loop-helix (bHLH)—periodic circadian protein (PER)—AhR nuclear translocator (ARNT)—single-minded protein (SIM) superfamily of transcription factors, which PER-ARNT-SIM (PAS) domain acts as a sensor of endogenous and exogenous factors (Nguyen and Bradfield, 2008). It is a primitive protein shared by all eumetazoan animals, suggesting its origin as c. 600 mya (Hahn et al., 2017). AhR is activated by a wide range of internal, external, and dietary compounds, metabolites of arachidonic acid and tryptophan, microbial byproducts, contaminants, and drugs that have been shown to bind to AhR with divergent affinities resulting in several different outcomes.

**FIGURE 1:**
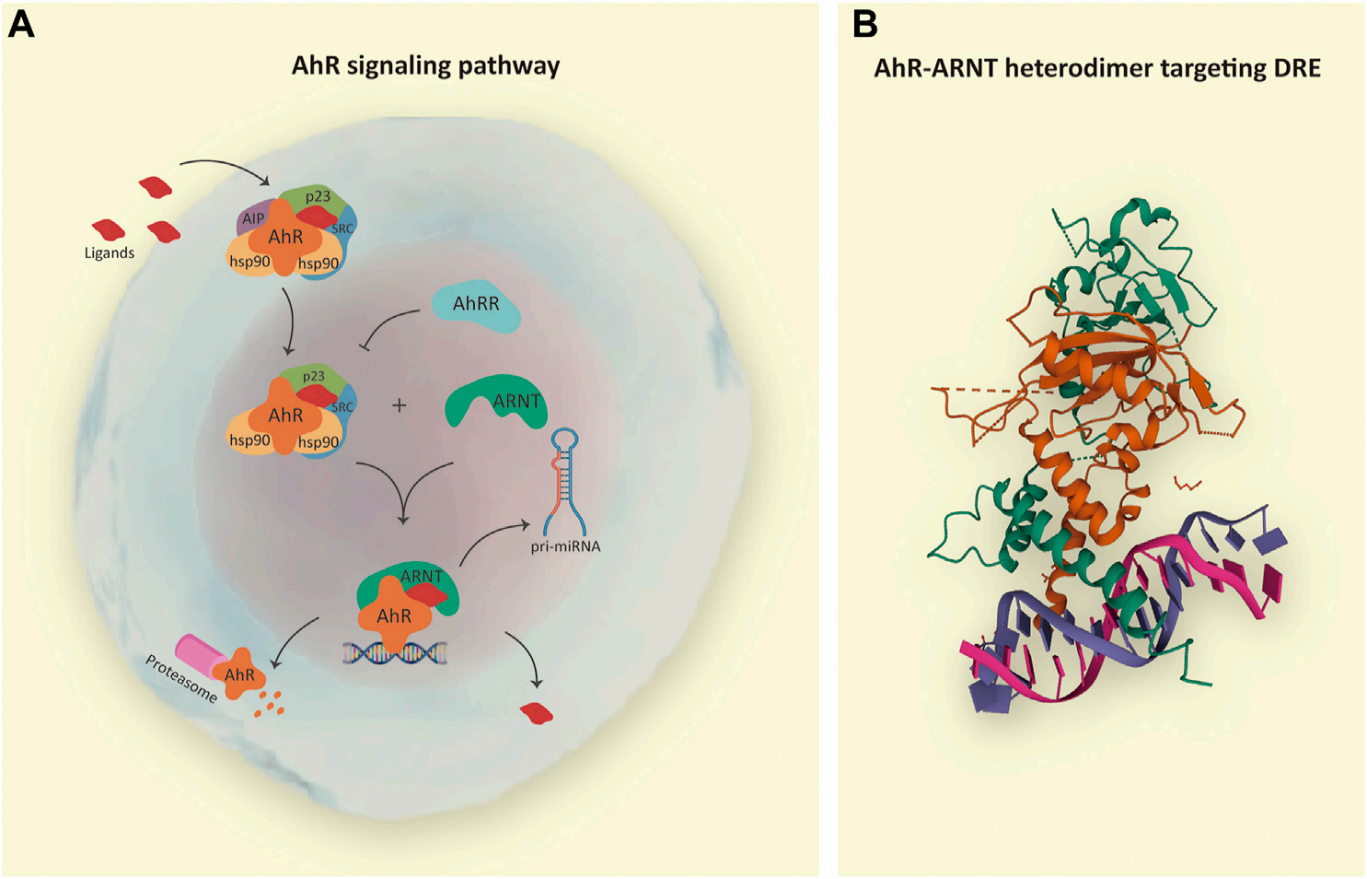
**(A)** AhR signaling pathway. The inactive form of AhR is localized in the cytosol complexed with chaperones: HSP90, AIP, p23, and SRC. Endogenous or exogenous AhR ligands enter the cell and induce conformational changes in AhR complex, resulting in AIP release and protein complex translocation to the nucleus in a transportin and importin-β-dependent manner. In the nucleus, AhR interacts with ARNT, and the heterodimer is responsible for the transcription of genes containing dioxin response elements (DRE), such as pri-microRNAs; whereas AhRR acts as a negative regulatory mechanism. After transcriptional regulation, the AhR-ARNT complex is withdrawn from DNA, and AhR is degraded in the cytosol by the proteasome. **(B)** In detail, crystal structure of the AhR-ARNT heterodimer in complex with the DRE (RCSB PDB—5V0L: x-ray diffraction method; resolution 4.00 Å; r-value free 0.323; r-value work 0.285; r-value observed 0.287; deposited by Seok et al., 2017). Notes: AhR, aryl hydrocarbon receptor; HSP90, heat shock protein 90; AIP, AhR-interacting protein; p23, cochaperone protein; SRC, tyrosine kinase protein; ARNT, aryl hydrocarbon receptor nuclear translocator; AhRR, AhR repressor; pri-miRNA, primary microRNA.

Naturally, AhR remains in the cytosol complexed with chaperone proteins, including 1) a dimer of the heat shock protein (HSP90); 2) the AhR-interacting protein (AIP; also known as immunophilin hepatitis B virus X-associated protein 2—XAP2); 3) the co-chaperone p23 (cytosolic prostaglandin E2 synthase); and 4) the protein kinase (c-SRC), which altogether help to keep the receptor in a high affinity for its ligands and stable in the cytoplasm preventing degradation and ubiquitination (Hoffman et al., 1991; Schrenk, 1998). Ligand binding results in a conformational change, dissociation of AIP from the complex exposing the AhR amino-terminal nuclear localization signal, an adjacent nuclear export signal, and a protein kinase C target site leading to its nuclear translocation in a transportin-dependent and importin-β-dependent manner. Ligand-independent activation of AhR has also been reported (Chang et al., 2007; Xiao et al., 2015; Lee et al., 2016) (Figure 1).

Nuclear AhR forms a heterodimer with AhR-nuclear translocator (ARNT, also known as HIF-1β) capable of binding cognate DNA dioxin response elements (DRE) and stimulating the expression of AhR target genes (Swanson et al., 1993; Schrenk, 1998; Huang et al., 2004; Huang and Elferink, 2012). The AhR repressor (AhRR), also induced by AhR, competes with the AhR-ligand complex for interaction with ARNT, limiting the ARNT availability and providing an additional negative regulatory mechanism (Sakurai et al., 2017). Also, hypoxia-inducible factor 1α (HIF-1α) has been shown to compete with AhR for its interaction with ARNT (Mascanfroni et al., 2015).

AhR prototypical regulated genes are the cytochrome P450 superfamily members CYP1A1, CYP1A2, and CYP1B1 (Nebert et al., 2004); the reason why, for almost 50 years, the AhR has been highlighted as a receptor for environmental contaminants and as a mediator of xenoprotective and drug-metabolizing genes (Poland et al., 1976).

In addition to xenobiotic metabolism and toxicity, new roles and multiple mechanistic pathways have been identified delineating how AhR activation leads to, for example, cell cycle control (Gasiewicz et al., 2014), hematopoiesis (Smith et al., 2013), organ development (Kiss et al., 2011), embryogenesis (Santiago-Josefat et al., 2001), and immunity (Kimura et al., 2009; Holloman et al., 2020). Moreover, the AhR is a pivotal effector in many pathological processes (Bock, 2020), such as cancer (Hanieh et al., 2016), intestinal inflammation (Lamas et al., 2016; Metidji et al., 2018), hepatic steatosis (Lee et al., 2010), chronic rhinosinusitis (Liu et al., 2018), and atopic dermatitis (van den Bogaard et al., 2013; Peppers et al., 2019).

AhR is mainly expressed in barrier organs such as skin, intestine, lung, and associated leukocytes (Barroso et al., 2021; Stockinger et al., 2021) and mediates functions in various immune cells controlling the differentiation of monocytes to dendritic cells (Goudot et al., 2017), T cells into regulatory T cells (Tregs), and Th17 cells (Nakahma et al., 2013; Singh et al., 2016; Al-Ghezi et al., 2019; Abdulla et al., 2021).

This collection of novel roles unrelated to pollutant assimilation is due to the AhR ability to regulate the activation of other DNA target sequences, co-activators such as toll-like receptors (TLRs) (Kado et al., 2017) or transcription factors as TGFβ/SMAD3 (Sarić et al., 2020), and, consequently, the transcriptional modules under their control (Tian et al., 2002). AhR seems to regulate innate inflammatory signaling also through direct binding to REL-A and REL-B, which are members of the NF-κB family of transcription factors (Øvrevik et al., 2014).

AhR-activated signaling pathways are also significantly associated with epigenetic modification (Li et al., 2011; Moret-Tatay et al., 2019). AhR modulates chromatin remodeling by controlling histone acetylation and methylation through poorly understood means. Additional mechanisms include controlling retrotransposons, long non-coding RNAs (ncRNA), and microRNAs (miRNA) (Garcia et al., 2017; Rogers et al., 2017; Miret et al., 2020). Just recently, AhR emerged as a suppressor of inflammation, oxidative stress, apoptosis, and immune regulator by miRNAs (Hecht et al., 2014; Chinen et al., 2015; Alzahrani et al., 2017). Additionally, studies have predicted that AhR 3′ untranslated region (UTR) may be targeted by miRNAs (Bleck et al., 2013; Liu et al., 2018), representing a prominent mutual control.

The miRNAs are a class of highly conserved, small, endogenous, and single-stranded ncRNA (∼18–25 nucleotides long) that control the expression of a large gene set at the post-transcriptional level. The miRNAs regulate mRNAs by directly binding at the 3′ UTR of the targets, usually resulting in their degradation or silencing (Bartel, 2004; O’Brien et al., 2018; Al-Ghezi et al., 2019). Animal miRNAs identify their targets using a small sequence known as *seed* (two to eight nucleotides) located at the 5′ end of the mature miRNA (Bartel, 2018).

Recent findings showed miRNAs playing a critical role in the regulation of gene expression and in the control of the majority of molecular and cellular pathways throughout eukaryotic organisms and also in viruses (Singh et al., 2016; Al-Ghezi et al., 2019). Multiple studies indicate that abnormal expression of miRNAs can control proliferation, apoptosis, maturation, and differentiation of immune cells (Singh et al., 2016; Lv et al., 2018; Al-Ghezi et al., 2019), as well as they represent a component of the innate immune responses that can restrain inflammatory signaling and interfere in different aspects of inflammation (Liu et al., 2018; Lv et al., 2018; Al-Ghezi et al., 2019; Disner et al., 2021; Pimentel Falcao et al., 2021).

As a potent transcription factor, AhR regulates the genetic expression of miRNAs and inversely might be regulated by them. According to Hecht et al. (2014), their study provided one of the first pieces of evidence for AhR expression to be essential for the physiological regulation of cellular miRNA levels. Likewise, Singh et al. (2016) demonstrated for the first time that the ability of AhR ligands to regulate the differentiation of Tregs vs Th17 cells might depend on miRNA signature profile. However, this is an emerging topic that demands further investigations. The limited number of research highlighting the connection between AhR and miRNAs is probably because of the still rising development of the field, where much effort has been made for mapping these molecules and annotating their biological functions, above all in diseases.

Here, we reviewed the interconnecting regulation of gene expression by miRNAs dependent on the activation of AhR driven by various ligands, and inversely the mechanisms that repress its activity modulated by miRNAs. We focused on novel pathways where AhR is involved beyond the environmental toxicity response, such as inflammatory response, metabolism, carcinogenesis, cellular differentiation and proliferation, and additional biological processes post-transcriptionally controlled by miRNAs after AhR modulation. In summary, this review attempts to integrate several biological aspects of AhR-mediated regulation of miRNAs, and vice versa, based upon the recent literature.

## The AhR Activation: Usually a Ligand Matter

TCDD is considered the prototype for studying the bioactivity of the AhR and historically is the most common ligand associated with AhR. TCDD is extensively used to study the impact of AhR activation on various physiological functions. Substantial studies have shown that the immune system is one of the most sensitive targets of TCDD (Hanieh and Alzahrani, 2013; Neamah et al., 2019; Abdulla et al., 2021).

Multiple mechanistic pathways have been identified to delineate how AhR activation leads to regulation of the immune system (Neamah et al., 2019). The evidence linking TCDD and miRNAs functions to human diseases is rapidly increasing, and miRNAs play an influential role in the toxicity of TCDD to animal models (Neamah et al., 2019). Also, some studies have confirmed an association between dysregulation of miRNAs and exposure to dioxin. It has been found that the toxic effects of TCDD may also be controlled by specific epigenetic mechanisms like DNA methylation or histone modification (Patrizi and Cumis, 2018).

In the literature, TCDD-dependent AhR activation has been implicated particularly in carcinogenesis and inflammatory responses. It is worth mentioning that inflammation is often associated with the development and progression of cancer. Zhang et al. (2012) reported that TCDD inhibited breast cancer cell invasion by inducing miR-335 and decreased SOX-4 (a miR-335-regulated gene) expression. Hanieh (2015) also presented similar findings regarding down-regulation of the pro-metastatic factor SOX-4 in breast cancer cells where TCDD triggered the AhR-miR-212/132 axis. The inhibitory effects of TCDD on the proliferation and invasion of prostate cancer cells were demonstrated by Yu et al. (2018); AhR enhanced the expression of miR-150-5p, which regulates MAP3K12. miR-150-5p might act as a suppressor of carcinogenesis once there is evidence of its low expression in prostate cancer tissues compared to non-tumorous control. Alzahrani et al. (2017) identify miR-132 as implicated in colitis-associated colon cancer (CAC) pathogenesis. In this study with mice, TCDD was used to activate AhR in induced CAC, which boosted miR-132 expression and alleviated CAC severity by suppressing macrophage infiltration, and pro-inflammatory cytokines and augmented a cholinergic anti-inflammation by inducing acetylcholinesterase (AChE)-targeting miR-132. This anti-inflammatory role is attributed mainly to targeting AChE that hydrolyzes acetylcholine (ACh), a neurotransmitter that suppresses pro-inflammatory cytokines by interrupting NF-κB nuclear translocation.

In the context of inflammation, Neamah et al. (2019) observed that naive C57BL/6 mice exposed to TCDD showed massive mobilization of myeloid-derived suppressor cells (MDSCs) in the peritoneal cavity and down-regulation of miRNA expression, notably miR-150-5p and miR-543-3p, that directly or indirectly enhance anti-inflammatory genes, including IL-10, PIM1, ARG2, STAT3, and CCL11. Additionally, Hanieh and Alzahrani (2013) found out that TCDD induced through AhR the miR-132, which attenuated experimental autoimmune encephalomyelitis (EAE). The TCDD also induced cholinergic anti-inflammation in EAE mice; overexpression of miR-132 in encephalitogenic CD4^+^ cells decreased IL-17 and IFN-γ and suppressed T-cell proliferation. Moreover, Al-Ghezi et al. (2019) investigated whether AhR activation by TCDD could attenuate the pertussis toxin-induced inflammation in mice. They found a significantly enhanced expression of miR-3082-5p that targets IL-17 and a decreased expression of miR-1224-5p, which targeted FOXP3, causing the generation of more Tregs but suppressing Th17 cells.

Further, little is known about the AhR regulation of miRNAs expression in the lung in response to inhaled toxicants. Cigarette smoke contains many toxicologically significant chemicals, including polycyclic aromatic hydrocarbons (PAH), and it is often studied since it represents one of the main risk factors for disease progression. Donate et al. (2021) found that cigarette smoke activates the AhR on Th17 cells directing the up-regulation of miR-132, which is then packaged into extracellular vesicles that induce osteoclastogenesis via the suppression of COX-2 that catalyzes prostaglandins. Rogers et al. (2017) also investigated the AhR-dependent regulation of pulmonary miRNAs by chronic cigarette smoke exposure and they have shown that AhR reduced the levels of miR-96, which is a potent suppressor of inflammation and strongly implicated in cancer progression.

**FIGURE 2:**
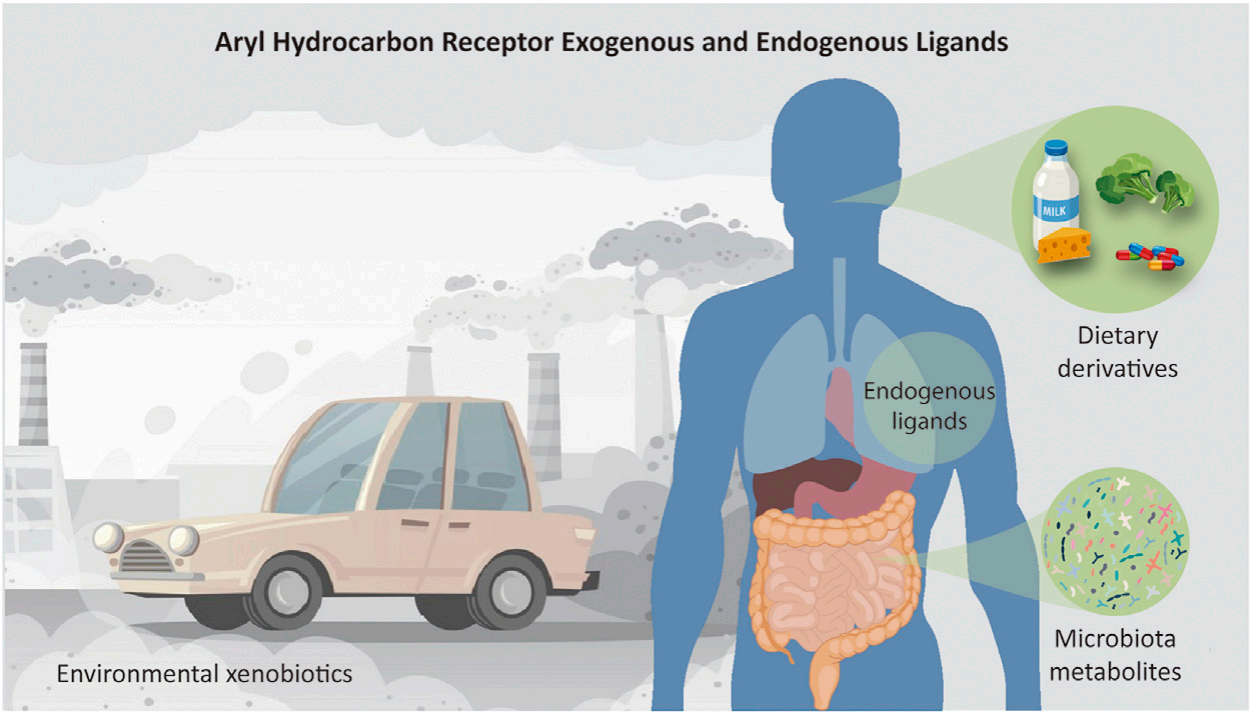
Schematic representation of all sources of ligands that activate the AhR receptor. AhR is a cytoplasm-located transcription factor that shuttles to the nucleus upon binding of a range of molecules. The ligands can be, for example, environmental chemicals such as 2,3,7,8-Tetrachlorodibenzo-p-dioxin (TCDD) or polycyclic aromatic hydrocarbons like Benzo(a)pyrene; plant-derived molecules such as indole-3-carbinol (I3C) and its acid catalyzed dimer, 3,3′-diindolylmethane (DIM), found in cruciferous vegetables; endogenous compounds, such as tryptophan-derived products like 6-formylindolo [3,2-b]carbazole (FICZ); products of commensal microbiota, e.g., indoles and naphthoquinone phthiocol; and medical drugs as omeprazole and tranilast. Conceived based on detailed lists of AhR ligands provided by literature.

Plenty of investigations have described other classes of AhR ligands, notably dietary composites and byproducts of microbiota metabolism (Figure 2). For example, the 3,3′-diindolylmethane (DIM), a dimer of indole-3-carbinol (I3C), is a naturally occurring dietary component found in cruciferous vegetables such as broccoli, Brussels sprouts, cabbage, and kale. The reputation of Brassica vegetables as healthy foods rests in part on DIM activities, which have been shown to possess anti-inflammatory, anticancer, and antioxidant properties.

Singh et al. (2016) tested DIM and I3C on mice with Delayed-type hypersensitivity response (DTH), proving their activities to be AhR-dependent, consequently leading to the mitigation of DTH response through the induction of anti-inflammatory Tregs and suppression of pro-inflammatory Th17 cells. This happened mainly due to the decreased expression of miR-31, miR-219, and miR-490 that target FoxP3, followed by the increased expression of miR-495 and miR-1192, which control IL-17 production. DIM effects have also been verified by Hanieh (2015) in the metastasis suppression through the control of SOX-4 by the miR-212/132 cluster; by Alzahrani et al. (2017) in the attenuation of tumorigenesis by miR-132; and Yu et al. (2018) in the AhR-miR-150-5p-MAP3K12 axis on cell proliferation and invasion of prostate cancer.

Furthermore, the literature has shown that the mode of action and responses mediated by AhR might be ligand-specific, cell-specific, and context-specific. It means that the number and kind of roles are potentially unlimited or at least far beyond what has been described so far. For instance, Abdulla et al. (2021) found that two conventional ligands [TCDD and 6-Formylindole (3,2-b)carbazole–FICZ] resulted in an opposite outcome in the context of DTH and especially in the miR-132 expression. While TCDD induced Tregs and attenuated the mBSA-mediated DTH response in mice, the FICZ induced Th17 cells and exacerbated this response. Similarly, Singh et al. (2016) described that the endogenous AhR ligand FICZ had precisely the opposite effects on the miRNAs involved in the induction of Tregs or Th17 compared to DIM and I3C.

In such a complex net of interactions, it is also possible that different ligands present a similar response, particularly in situations where the context might impose a greater contribution. For example, in two studies, Hanieh, (2015; 2016) tested different ligands (TCDD, DIM, and favipin–a non-toxic flavonoid) and found similar outcomes in human breast cancer tumorigenesis. These treatments on MDA-MB-231 and T47D breast cancer cells led to the AhR-regulated overexpression of the miR-212/132 cluster, which, in turn, reduced migration, invasion, and metastasis through suppressing the pro-metastatic transcription factor SOX-4 that present binding sites for the miRNA cluster. This is quite interesting once it reinforces AhR as a promising target to control migration and invasion in breast cancer cells through *in vitro* preliminary tests, and suggests the possibility to use less or non-toxic AhR ligands that leads to similar responses. However, there would be limitations to achieve high enough concentration to reproduce such events in whole animals. Other classes of ligands and their involvement in different biomolecular responses over experimental models can be checked in Table 1.

**TABLE 1:** Summary of the reviewed papers examining AhR-miRNA axis mutual regulation pathways.

Context	Ligand	Model	miRNAs	Highlights	References
Inflammation	N/A	Mice with colitis	miR-212/132	miR promotes inflammatory response by > Th17 cells; <IL-10 producing T cells; vital role of AhR in intestinal homeostasis	Chinen et al. (2015)
Inflammation	TCDD	EAE mice	miR-132	Targeting AChE attenuates EAE by cholinergic anti-inflammation; >miR suppresses T cell proliferation, IL-17 and IFN-y; while < IL-6, IL-1β, and TNF-α	Hanieh and Alzahrani (2013)
Cell cycle	N/A	EAE mice	miR-212/132	Promotes IL-17-producing Th cell differentiation	Nakahama et al. (2013)
Tumorigenesis	Flavipin	MDA-MB-231 and T47D BC cells	miR 212/132	Suppress breast cancer cells metastasis and invasion controlling SOX4	Hanieh et al. (2016)
Tumorigenesis	TCDD; DIM	MDA-MB-231 and T47D cells	miR 212/132	Suppress metastasis targeting the pro-metastatic factor SOX4	Hanieh (2015)
Tumorigenesis	TCDD; DIM	Mice with colitis	miR-132	Attenuates tumorigenesis and augmented a cholinergic anti-inflammation by targeting AChE	Alzahrani et al. (2017)
Inflammation	TCDD; FICZ	C57BL/6 mice	miR-132	TCDD upregulated miR and induced Tregs, attenuating response; FICZ had an opposite effect and induced Th-17 cells	Abdulla et al. (2021)
Inflammation	CS	Mice, mice immune cells; RA patients	>miR-132 and miR-346	<COX2 that catalyzes prostaglandins, inducing murine osteoclastogenesis. RA patients who smoke express just a higher level of miRNA-132, not miR-346.	Donate et al. (2021)
Tumorigenesis	TCDD; DIM	Prostate cancer	miR-150-5p	Suppress prostate cancer progression by regulating MAP3K12	Yu et al. (2018)
Inflammation	TCDD	C57BL/6 mice	< miR-150-5p and miR-543-3p	The miRs target and enhance anti-inflammatory and regulatory genes, including IL-10, PIM1, ARG2, STAT3, and CCL11	Neamah et al. (2019)
Inflammation	TCDD	C57BL/6 mice	> miR-3082-5p (1)	Target IL-17 (1); target FOXP3 (2), generating anti-inflammatory Tregs but suppressing pro-inflammatory Th17 cells attenuating inflammatory response	Al-Ghezi et al. (2019)
			< miR-1224-5p (2)		
Inflammation	I3C; DIM; FICZ	Mice with DTH	<miR-31; miR-219; miR-490 (1)	>FOXP3; suppresses DTH response through induction of Tregs and suppression of Th17 cells	Singh et al. (2016)
			>miR-495; miR-1192 (2)	(1); <IL-17 (2)	
Inflammation	Alpinetin	Mice with colitis	miR-302; miR-148a	Treg differentiation but exerted little effect on Th17 differentiation	Lv et al. (2018)
Cell cycle	Tranilast	MEF cells	miR-302	Contributes to maintenance of pluripotency and cell reprogramming	Hu et al. (2013)
Tumorigenesis	TCDD; MCDF	MDA-MB-231 and BT474 cells	miR-335	<SOX-4; inhibits breast cancer cell invasion and metastasis	Zhang et al. (2012)
Toxicity	Oltipraz	Mice	miR-125b	<AhRR; protect kidney from cisplatin-induced acute injury	Joo et al. (2013)
Experimental	N/A	HUH-7 and HepG2 cells	miR-24	<ARNT post-transcriptionally	Oda et al. (2012)
Tumorigenesis	Berberine	MCF-7 cells	miR-21-3p	<CYP1A1 post-transcriptionally	Lo et al. (2017)
Tumorigenesis	N/A	Neuroblastoma tumor	miR-124	<AhR, responsible for promoting neuroblastoma SK-N-SH cell differentiation, cell cycle arrest, and apoptosis.	Huang et al. (2011)
Inflammation	N/A	IECs and mice with colitis	miR-124	miR up-regulates the expression of pro-inflammatory cytokines through suppressing AhR	Zhao et al. (2016)
Inflammation	N/A	Nasal polyps in chronic rhinosinusitis	miR-124	<AhR; <TNFα	Liu et al. (2018)
Inflammation	N/A	Macrophages from RA patients	miR-223	<ARNT and increases cytokine production	Ogando et al. (2016)
Toxicity	Acetaminophen	Mice with ALF; Human hepatocytes	miR-122	<AhR; < CYP1A2 expression; modulates positively drug toxicity	Chowdhary et al. (2017)
Inflammation	PM; DEP	pHBEC	miR-375	<AhR	Bleck et al. (2013)
Tumorigenesis	N/A	MDA-MB-231 and MCF-7 cells	miR-548m	<AhR	Wm Nor et al. (2021)
Metabolic disorders	Ginger-derived nanoparticles	C57BL/6 mice	miR-375	<AhR	Kumar et al. (2021)
Osteoporosis	kynurenine	BMSC cells	miR-29b-1-5p	>AhR; feedback mechanism exists between them	Elmansi et al. (2020)
Toxicity	B(a)P	Primary hepatocyte culture; Rat livers	miR-483-3p	Increased oncogenic miR levels	Filippov et al. (2019)
Occupational exposure	HpCDD	Human Lung Fibroblast	let-7d-5p; miR-103-3p; miR-144-3p; miR-107	miRs as biomarkers to chemical exposure to hazardous agents	Woeller et al. (2019)
Inflammation	CS	Mice	miR-96; miR-34c; miR-146a; miR-135b; miR-196a	<miR-96 may increase FOXO3a and be how AhR attenuates inflammation; the miR is also implicated in cancer progression.	Rogers et al. (2017)
Tumorigenesis	N/A	Human endometrium tumor cells	miR-28; miR-30c; miR-30e; miR-139; miR-153	miRs reduced in tumors (2-3-fold), confirming the oncosuppressor-related activity	Ushakov et al. (2018)
Cell cycle	Ligand-independent	Mouse lung fibroblast	miR-196a	Controls lung fibroblast apoptosis, but not proliferation.	Hecht et al. (2014)
Inflammation	I3C	C57BL/6 mice	<miR-29b-2-5p	Increased IL-22 expression, ameliorating lung injury	Holloman et al. (2020)

Notes: AhR, aryl hydrocarbon receptor; AhRR, AhR repressor; ALF, acute liver failure; ARNT, aryl hydrocarbon receptor nuclear translocator; B(a)P, benzo(a)pyrene; BMSC, bone marrow mesenchymal stem cells; CS, cigarette smoke; DEP, diesel exhaust particles; DIM, 3,3’-diindolylmethane; DTH, delayed-type hypersensitivity response; EAE, autoimmune encephalomyelitis; FICZ, 6-formylindole(3,2-b)carbazole; HpCDD, heptachlorodibenzo-p-dioxin; I3C, indole-3-carbinol; IEC, intestinal epithelial cells; MCDF, 6-methyl-1,3,-trichlorodibenzofuran; N/A, not applicable; pHBEC, primary human bronchial epithelial cells; PM, ambient particulate matter; RA, rheumatoid arthritis; TCDD, 2,3,7,8-tetrachlorodibenzo-p-dioxin.

Although AhR ligand activation is quite established in miRNA expression changes, whether the AhR contributes to miRNA levels independent of xenobiotics is not deeply explored. The literature has also been unpretentiously portraying this receptor as ligand-independent in some circumstances, especially where the pathological condition or the situation evaluated are the reason to make the receptor-activated and responsive to the stressor situation. Hecht et al. (2014) reported for the first time that the AhR potently regulates miRNA levels in pulmonary cells, and in particular, promotes the expression of miR-196a in the absence of xenobiotics. This opens up the possibility of a critical feature in the ability of the AhR to exert physiological control independent of exogenous ligands. The authors confirmed that AhR ligation with benzo(a)pyrene [B(a)P], FICZ, or CH-223191 reduced AhR protein expression concomitant with decreased miR-196a levels. However endogenous ligand cooperation or intrinsic mechanisms cannot be ruled out. Still, other pieces of evidence unrelated to epigenetic regulation also demonstrated ligand-independent activation of AhR (Xiao et al., 2015; Lee et al., 2016).

## AhR-Regulated miRNAs

It has been suggested that the epigenetic processes might be based on the AhR-mediated transcription of the target miRNA genes that have the DREs in their promoters, that lead to different health problems and disease progression (Hecht et al., 2014; Al-Ghezi et al., 2019; Filippov et al., 2019; Abdulla et al., 2021). In the past decade, miRNAs have emerged as major players in cellular modulation. Since they have such pivotal roles in gene regulation, it is crucial to understand the relationship of miRNAs with diseases and biological processes.

The conserved miR-212/132 cluster has appeared as the most frequent miRNA associated with AhR activation throughout our review. Chinen et al. (2015) have shown that it had an elevated expression in the colon of wild-type (WT) mice with colitis, an inflammatory condition of the colon inner lining. In this context, the AhR-miR-212/132 axis promotes inflammatory response by inducing Th17 cells and suppressing the development of IL-10-producing T cells, which maintains intestinal homeostasis. Additionally, according to the authors, the group had previously reported that these two miRNAs, when induced by AhR, promote Th17 cell differentiation by inhibition of the B-cell lymphoma 6 protein (Bcl-6, a transcription repressor), suppression of IL-1β and IL-6 mRNA expression, and negatively regulate IL-6 production. Similarly, Nakahama et al. (2013) demonstrated an essential role of the cluster in the context of EAE. miR-212/132 deficient mice exhibited significantly higher resistance to the development of EAE and lower frequencies of both Th1 and Th17 cells in draining lymph nodes.

Also, Hanieh and Alzahrani (2013) have shown that miR-132 attenuates EAE by inducing cholinergic anti-inflammation by up-regulating AChE targeting miR-132. This miRNA overexpression in encephalitogenic CD4^+^ cells decreased IL-17 and IFN-γ and suppressed T-cell proliferation. In EAE mice, AhR activation resulted in similar expression profiles between miR-212 and miR-132. However, miR-132 is more relevant to the central nervous system (CNS) because it is highly expressed in the brain.

The activation of AhR by TCDD attenuated DTH response in mice and caused up-regulation of miR-132, as demonstrated by Abdulla et al. (2021). Transfection studies revealed that miR-132 targeted High Mobility Group Box 1 (HMGB1), whose down-regulation caused an increase in FoxP3^+^ Treg differentiation and suppression of Th17. Moreover, this response was less effective in miR-132 deficient mice. However, in the same research, when using FICZ as the ligand, the authors reported the miR-132 down-regulation and an augmented DTH response.

Donate et al. (2021) have shown that miR-132 can be induced in Th17 and acts as a pro-inflammatory mediator through down-regulation of *Ptsg2* expression and COX-2 transcription, which contributes to the development and progression in experimental and clinical rheumatoid arthritis (RA), mainly by exacerbating local inflammation and joint destruction.

Moreover, Hanieh, (2015; 2016) assessed the AhR-miR-212/132 axis in breast cancer cell lines and confirmed the anti-metastatic properties of the miRNA cluster through suppression of SOX-4, which inversely is a pro-metastatic factor and has binding sites for miR-212/132 on the 3′ UTR region. Last but not least, Alzahrani et al. (2017) also found that enhanced miR-132 expression by AhR reduced the colon cancer tumorigenesis associated with chronic inflammation. Overall the surveyed data points to the miR-212/132 cluster as a viable therapeutic subject, once it might reduce the inflammation and attenuates oncogenesis.

Additionally, miR-302 was exhibited as another miRNA regulated by unusual AhR ligands and having different cellular effects. miR-302 is a polycistronic miRNA cluster including miR-302a/b/c/d and miR-367. Particularly miR-302 is highly expressed in embryonic stem cells (ESC). On account of the pivotal role of miR-302 in the maintenance of pluripotency and the cell reprogramming process, Hu et al. (2013) screened for prospective chemicals to manipulate its expression. They found that tranilast, an anti-allergy drug, can significantly up-regulate miR-302 by activating AhR, promoting somatic cell reprogramming in a miR-302-dependent way. Lv et al. (2018) have shown that alpinetin, a flavonoid compound extracted from *Alpinia katsumadai hayata* seeds, regulates miR-302 and ameliorated colitis in mice via promoting Treg differentiation in an AhR-dependent manner but exerted little effect on Th17 differentiation.

Furthermore, notwithstanding habitual measurement of CYP1A1 regulation, a downstream target gene, to confirm the AhR functionality, Lo et al. (2017) found that berberine, a protoberberine alkaloid present in several medicinal plants, such as goldenseal (*Hydrastis canadensis*), activates AhR but, on the other hand, suppresses CYP1A1 protein expression through the miR-21-3p stimulation in MCF-7 breast cancer cells, which might be a feedback control. Berberine significantly increased miR-21-3p by 36%, and the transfection of a miRNA inhibitor restored the induction of CYP1A1 protein with a 50% increase. However, the increase of CYP1A1 protein by berberine was only ∼16% of that by TCDD, which might be the most potent existing ligand, but extremely toxic.

The miR-150-5p is an interesting example of a tumor suppressor miRNA. Yu et al. (2018) revealed that AhR enhances the expression of miR-150-5p to suppress cell proliferation and invasion in prostate cancer by regulating MAP3K12.

**FIGURE 3:**
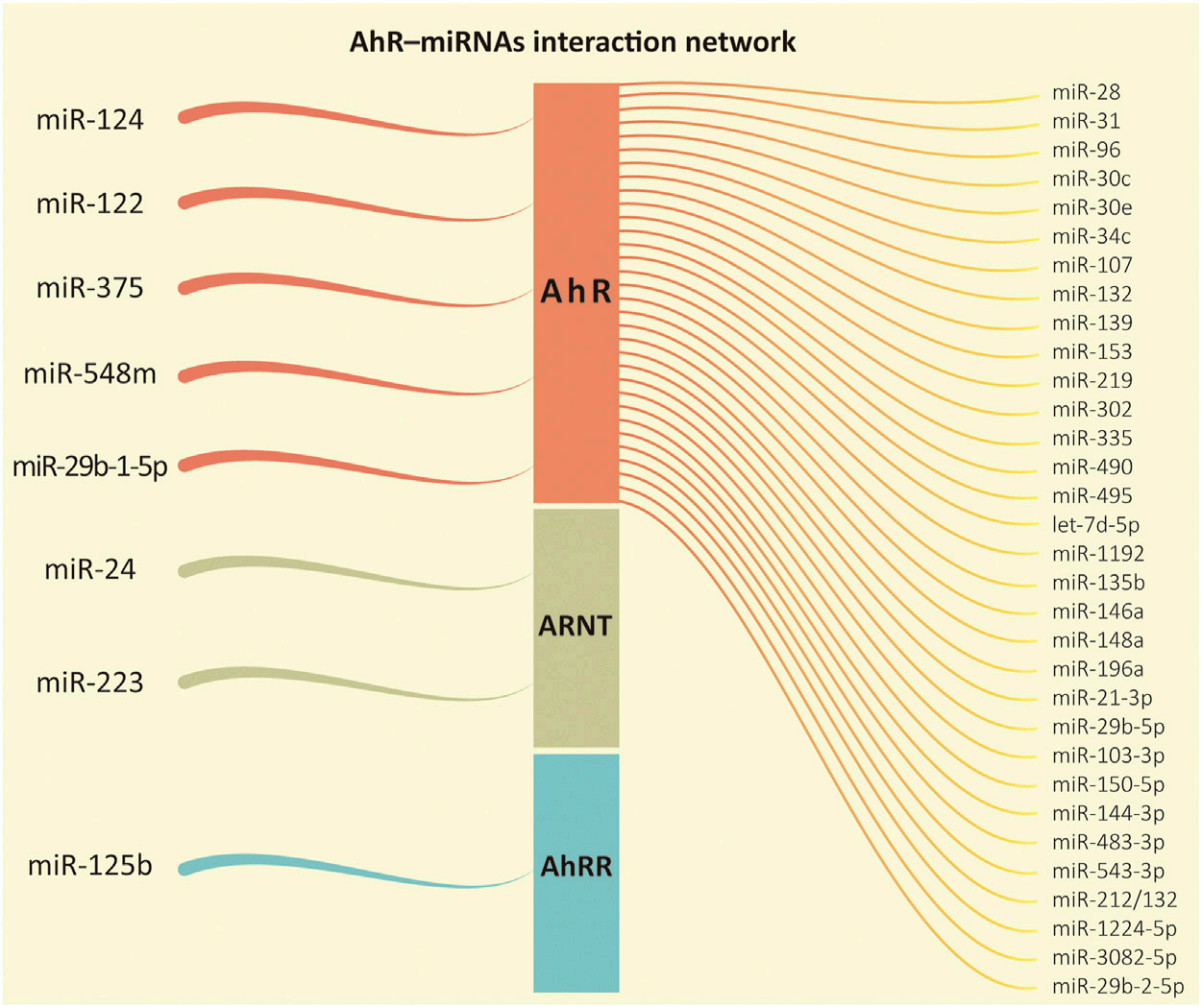
AhR-miRNAs interaction network. From left to right, it represents the microRNAs whose functions were recorded regulating one of the proteins in the aryl hydrocarbon receptor (AhR) signaling pathway followed by the miRNAs regulated downstream by the transcription factor AhR according to our review.

Briefly, it is not always true that AhR activates the expression of the miRNAs under its control. As presented in the examples above, it is also possible that the miRNAs have their levels diminished in the cell in certain conditions. This pattern was also reported in mice by Al-Ghezi et al. (2019) to miR-1224-5p; Singh et al. (2016) to miR-31, miR-219, and miR-490; Rogers et al. (2017) to miR-96; and Holloman et al. (2020) to miR-29b-2-5p. Other examples of miRNAs under AhR control or regulating the AhR pathway are shown in Figure 3 and Table 1.

## AhR and miRNAs: Tit for Tat

The majority of the evidence reviewed so far has pointed out that AhR often up-regulates the expression of several miRNA genes. Despite that, *in silico* analysis indicated that AhR might contain binding sites of miRNAs as well, which could demonstrate a regulation in the reverse direction (Figure 3).

According to a TargetScan examination performed by Liu et al. (2018), the miR-124ab family, which is highly conserved among humans, mice, and most species, has the putative ability to control AhR levels. Moreover, the authors exhibited that miR-124 could modulate cellular inflammatory response through negatively regulating AhR expression and inflammatory cytokine (TNF-α) that is critical to the development of inflammatory response in chronic rhinosinusitis (CRS) with nasal polyps (CRSwNPs). Besides, the AhR is also regulated by TGF-β, whose biological activities are shared with the AhR (e.g., proliferation, apoptosis, among others; Hecht et al., 2014).

miR-124 also suppressed AhR in colon tissues and intestinal epithelial cells (IEC) of active Crohn’s disease (CD) patients. Zhao et al. (2016) suggested that miR-124 has a pro-inflammatory role in intestinal inflammation by inhibiting AhR and modulating pro-inflammatory cytokines production. They found an inverse correlation between miR-124 and AhR protein levels in colon tissues and IECs of CD patients and further demonstrated this interconnection in Caco-2 and HT-29 cells.

Elsewhere, the miR-124 does not act just in the inflammatory response, it is also an abundant neuronal miRNA. Huang et al. (2011) showed it is plausible that miR-124 may affect neuroblastoma once knockdown of miR-124 induces neuroblastoma SK-N-SH cell differentiation, cell cycle arrest, and apoptosis through promoting AhR. Neuroblastoma is a type of cancer that forms from immature nerve cells, most frequently in one of the adrenal glands.

Still, in the tumorigenesis context, Wm Nor et al. (2021) demonstrated that miR-548m modulates epithelial-mesenchymal transition (EMT) in two breast cancer cell lines, MDA-MB-231 and MCF-7. Although the miR-548m molecular mechanisms and associated functions in cancers have not been fully elucidated, its expression has been found in various types of cancers, and the authors hypothesized it might act as a tumor suppressor since its overexpression in both cell lines increased E-cadherin expression and decreased the EMT-associated transcription factors, significantly inhibiting migration and invasion capabilities. In addition, the study identified AhR as a direct target of miR-548m and suggested a novel function in reversing the EMT by decreasing their migratory and invasive potentials, at least in part *via* targeting AhR expression.

On the other hand, miR-122 is a liver-specific conserved miRNA that regulates metabolic homeostasis. Chowdhary et al. (2017) found that miR-122 is down-regulated in liver biopsy specimens of patients with acute liver failure (ALF) and acetaminophen (paracetamol) treated mice, suggesting that acute liver injury suppresses miR-122 expression since it protects mice and human hepatocytes from acetaminophen toxicity by regulating cytochrome P450 family members expression. To determine whether miR-122 can modulate AhR and CYP1A2 expression, they transfected miR-122^−/−^ hepatocytes in culture with miR-122 mimic and confirmed that ectopic miR-122 suppressed AhR and CYP1A2 RNA levels.

In a different scenario, Bleck et al. (2013) provided data supporting the link between miR-375 and AhR expression in airway epithelial cells treated with environmental pollutants. The treatment of primary human bronchial epithelial cells (pHBEC) with diesel exhaust particles (DEP) or fine particulate matter (PM) increased miR-375, as well as thymic stromal lymphopoietin (TSLP), suggesting the presence of an intermediary signal that miR-375 might down-regulate. *In silico* evaluation suggested AhR as one possible target of miR-375. To confirm this interaction, the authors examined the influence of mimic miR-375 on AhR, whose transfection resulted in a small but significant down-regulation of AhR mRNA compared with resting levels in pHBEC. Whereas AhR was decreased in pHBEC treated with DEP, AhR transcripts increased when treated with DEP after transfection with anti-miR-375. So, they demonstrated that air pollutants up-regulated human miR-375 and TSLP in pHBEC cells and suggested AhR as an intermediary in this pathway.

Moreover, Kumar et al. (2021) reported that miR-375 targets AhR in mice while the authors intended to assess the molecular mechanisms underlying ginger-derived nanoparticle (GDNP)-mediated prevention of high-fat diet (HFD)-induced insulin resistance. In the study, induction of intracellular miR-375 led to inhibition of AhR expression and VAMP7, which has been suggested to be involved in exosome biogenesis. The findings indicate that VAMP7 monitors intracellular levels of miR-375 to prevent an uncontrolled reduction of AhR by sorting miR-375 into exosomes. Thus, the process restores AhR homeostasis expression disrupted by an HFD.

Additionally, Elmansi et al. (2020) described three related mechanisms (miR-29b-1-5p, CXCL12, and Hdac3) through which kynurenine, a tryptophan metabolite, affects bone marrow stromal cells in aging, all mediated through binding and nuclear translocation of the xenogeneic AhR. The authors suggested that either miR-29b-1-5p is upstream of AhR activation, or a feedback regulation mechanism exists between both. Categorically, it was demonstrated that miR-29b-1-5p mimic up-regulates AhR transcriptional level, and the antagomir down-regulates it.

Similarly, other researchers intended to investigate whether miRNAs could also regulate ARNT. Previous studies pointed out that the ARNT protein level was decreased by reactive oxygen species (ROS) or hydrogen peroxide (H_2_O_2_) in human cell lines (Choi et al., 2008). According to Oda et al. (2012), overexpression of miR-24 in HuH-7 and HepG2 cells significantly decreased the ARNT protein level but not its mRNA level, indicating translational repression. Such miR-24-dependent down-regulation concomitantly diminished downstream gene expression, like CYP1A1 and carbonic anhydrase IX. Two other miRNAs predicted by *in silico* analysis (miR-16 and miR-23b) were also investigated by these authors; however, their overexpression caused no change in the ARNT signature.

Likewise, Ogando et al. (2016) compared miRNA expression in CD14^+^ cells from patients with active osteoarthritis (OA) or RA. The analysis identified miR-223 as the only miRNA up-regulated in RA-derived cells. Their results associate Notch signaling to miR-223 down-regulation in RA macrophages and identify miR-223 as a negative regulator of the AhR/ARNT pathway through ARNT targeting.

Finally, Joo et al. (2013) have portrayed a nuclear factor erythroid-2-related factor 2 (Nrf2) and AhR crosstalk representing an indirect pathway in which miRNAs can interfere with AhR functionality targeting the AhRR. Specifically, the authors have shown that miR-125b is transactivated by Nrf2 and inhibits AhRR. miR-125b was the only investigated miRNA with increased expression in mice kidneys treated with oltipraz (an Nrf2 activator). Then, this integrative network protected the kidney from cisplatin toxicity through miR-125b up-regulation, which targeted AhRR leading to AhR activation.

## Novel Functions for an Old System: Advancements and Perspectives

Primary arguments concerning physiological roles for AhR beyond detoxification derive from the phenotypic evaluation of AhR^−/−^ mice (Fernandez-Salguero et al., 1995). AhR-deficient models exhibited several morphological defects, supporting the involvement of the protein in differentiation and development. The zebrafish (*Danio rerio*) is another well-established vertebrate model used to untangle the AhR pathway (Shankar et al., 2020). In contrast to mammals, which express a single AhR gene, zebrafish exhibits three AhR genes (*ahr1a*, *ahr1b*, and *ahr2*) due to a pair of whole-genome duplication (WGD) events in early vertebrate lineage followed by an additional teleost-specific WGD that happened about 350 mya (Glasauer and Neuhauss, 2014). The AhR features were also noticed in non-vertebrate models. In Drosophila, AhR orthologs (spineless) are involved in the legs and antenna morphogenesis and, in *Caenorhabditis elegans*, the ortholog (AhR1) seems to be required for the migration and differentiation of GABAergic neurons (Emmons et al., 1999; Huang et al., 2004). Interestingly, there is no evidence for the orthologs contribution to xenobiotic detection in these species since they appear to present mainly developmental functions (Hahn, 2002; Barouki et al., 2012).

Current research has been depicting AhR as far more than an environmental sensor. As stated in our review, this receptor’s adaptive functions also contribute to regulatory pathways that are not simply related to chemical exposure, remarkably in immune-mediated inflammatory diseases (IMIDs) and oncogenesis. Both disorders are characterized mainly by altered immune regulation causing chronic inflammation and disruption of cell signaling, disordered proliferation, and invasion in target organs and systems. Nonetheless, xenobiotics could either intensify the endogenous AhR function or antagonize it. New evidence for physiological functions of AhR emerged, primary for the immune system and later for the nervous system, maintenance of barrier organs, metabolic regulation, host-microbiome interactions, and oncogenesis (Esser et al., 2018).

Thus, AhR contribution to the immune system appears to be of primary importance. It is believed that AhR could be involved in autoimmunity, inflammation, and defense against infections (Barouki et al., 2012). AhR has been demonstrated to play a fundamental role in regulating Th17/Treg balance (Lv et al., 2018). Regarding the inflammatory pathways targeted by miRNAs, a few examples include miR-1224-3p and miR-142-3p that targeted the TGF-β pathway; miR-3082-5p that targeted IL-17; and several miRs (miR-671-5p, miR-505-5p, miR-27a-5p, miR-5112, miR-146a-5p) that targeted IL-10 (Al-Ghezi et al., 2019).

AhR also interferes in cancer progression. Results from experiments in mice that overexpress a constitutively active AhR (Andersson et al., 2002) showed that these animals are more prone to develop cancers. In contrast, immortalized mouse embryo fibroblasts with deleted AhR genes exhibit less tumorigenicity than WT in xenograft models (Mulero-Navarro et al., 2005).

Many miRNAs are involved in the pro-metastatic activity or as tumor suppressors (e.g., miR-483-3p and miR-139, respectively). Namely, Filippov et al. (2019) confirmed the existence of the AhR-mediated pathway in the expression regulation of miR-483-3p under B(a)P exposure in the primary culture of hepatocytes and liver of Wistar rats. The miR-483-3p was correlated to the toxicity of this potent PAH once its levels increased under treatment. Similarly, the expression of miRNAs indicated in the literature as oncosuppressors were studied in endometrium malignant tumors by Ushakov et al. (2018). Previous bioinformatic analysis has detected that miR-28, miR-30c, miR-30e, miR-139, and miR-153 are likely regulated by AhR. The tumor suppression function was confirmed because their relative expression was 2 to 3-fold below the normal in the endometrial samples. Another example is miR-150-5p, which down-regulation was proved to be a poor prognostic factor for prostate cancer patients (Yu et al., 2018).

Similarly, miRNAs may work as exposure biomarkers. The concept that these molecules can recall occupational exposure to hazardous environmental agents, precisely PAHs, polychlorinated dibenzo-p-dioxins (PCDDs), and polychlorinated dibenzofurans (PCDFs) is present in the Woeller et al. (2019) investigation. This is mainly due to, for instance, miRNAs’ longer persistence in serum than PAHs. According to the authors, benzo (ghi)perylene (BghiP) and heptachlorodibenzo-p-dioxin (HpCDD) are elevated in case-post-deployment serum samples after airborne occupational exposure related with open fire pit-combustion of residues. In this study, human lung fibroblasts (HLF) were exposed to BghiP and HpCDD (the latter is closely related to TCDD). It was not observed significant changes in HLF miRNA levels using BghiP exposure. On the other hand, HpCDD-mediated changes in let-7d-5p, miR-103-3p, miR-144-3p, and miR-107 in an AhR-dependent manner.

Not all AhR induction is associated with toxicity, as many ligands are used as therapeutic agents to induce the transcription factor activity. As a matter of fact, the employment of miRNAs as biopharmaceuticals targeting pathways of human disease is a promising distinctive feature that provides a new and potentially strong candidate for next-generation medicine. RNA-based biopharmaceuticals signify a new area for drug discovery and development. It has been estimated that its market value was worth about $1.2 billion at the end of 2020 (Bansal et al., 2016; Chakraborty et al., 2021). In practice, the ability of meticulously selected miRNAs to target altered mRNAs in pathologies turns these molecules into powerful candidates as therapeutics (through miRNA mimics) or as targets of therapeutics (as antimiRs) (Rupaimoole and Slack, 2017).

Similarly, miRNAs are interesting therapeutic tools for IMIDs, a range of autoimmune inflammatory disorders distinguished by a rupture in cellular homeostasis through multifactorial causes. This class of diseases affects around 3–7% of the population and may cause tissular injury of the target organ because of the uncontrolled inflammatory reaction. Thus, the autoreactivity phenomenon is usually observed due to the immune system’s uncontrollable production of antibodies triggered by the loss of tolerance to autoantigens and environmental antigens (Chen et al., 2019; García et al., 2020). Some examples discussed herein include inflammatory bowel diseases (e.g., Crohn’s disease), rheumatoid arthritis, and multiple sclerosis (MS).

## Final Remarks

All things considered, several of the studies reviewed here stated that their findings demonstrate for the first time a specific activity exerted by AhR involving miRNAs. It is symbolically exemplified by Rogers et al. (2017), after which exposure to cigarette smoke caused a dramatic increase in the number of miRNA in the lungs of both AhR^−/−^ and AhR^+/−^ mice, which clearly shows the recent discovery of this mutual involvement in such sensitive processes.

Considerable evidence strengthens the AhR as a distinctive transcription factor with crucial physiological roles in developmental origins of health and disease. This can be considered a paradigm shift since there are multiple pathways to be taken by the receptor even in the absence of xenobiotics, reinforcing its novel roles apart from the environmental sensor function.

Meanwhile, there are great expectations for the extended use of responsive models, such as zebrafish, not just regarding its AhR functions but also the regulatory network they exert with miRNAs. Notably because zebrafish has its genome fully sequenced, a considerable amount of annotated miRNAs, a high homology with humans, and can mediate the drug response in a correspondent manner to higher models, increasing its translational value.

Although it remains an important and unsolved question about all the varying manners in which AhR-dependent gene expression can occur, it is about time to apply the knowledge of AhR signaling to generate AhR-targeted therapeutics. It is noteworthy the numerous preclinical studies utilizing various disease models that have tested the use of these new-generation therapies and several miRNA-based therapeutics have advanced into clinical testing. As more miRNAs are applied in large-scale validation approaches and clinical trials, we get closer to effective biomarkers turning into a reality. On the whole, this is why it is crucial to deepen the knowledge about such processes in the largest possible number of experimental disease models.

In conclusion, these findings will guide future research efforts and reveal novel opportunities for AhR- and miRNA-targeted therapeutics.
